# S100A8 regulated by estrogen improves injured endometrial epithelium reconstruction by promoting tight junction formation and stromal cell transformation

**DOI:** 10.1038/s41598-025-08530-0

**Published:** 2025-07-01

**Authors:** Xiaodan Li, Fang Jia, Huiwen Bai, Xuejing Chen, Yue Zhang, Ningfang Zhang, Hongxin Yang

**Affiliations:** 1 College of Basic Medicine, Inner Mongolia Medical University, Hohhot, China; 2 First Clinical Medical College, Inner Mongolia Medical University, Hohhot, China

**Keywords:** Endometrial injury, Epithelium reconstruction, S100A8, Estrogen, Cell junction, Transformation, Cell biology, Diseases, Molecular medicine

## Abstract

Estrogen is used for endometrial repair; however, it has limited effectiveness. Cytokine S100A8 expression is affected by estrogen and is involved in regulating the damage repair. We aimed to confirm the effects of S100A8 and explore its mechanisms during the reconstruction of the injured endometrium. We investigated the effects of estrogen on S100A8 expression in healthy endometrium. A rat model of endometrial injury and cultured primary endometrial cells were used to determine the pleiotropic effects of S100A8 on endometrial epithelial repair. Estrogen regulated the recruitment of S100A8-positive immune cells (S100A8-PICs) and the release of S100A8 in the healthy endometrium, but estrogen plays a limited role in regulating seriously injured endometrium via self-S100A8. S100A8 administration to the uterine cavity significantly improved the morphology of injured epithelium; influenced the reverse migration of S100A8-PICs; and promoted epithelial localization of proliferating cells, cell junction formation, and transformation of stromal cells to epithelial cells. S100A8 in the uterine cavity has pleiotropic effects that improve endometrial epithelial reconstruction and can compensate for the deficiency in estrogen repair regulation.

**Supplementary Information:**

The online version contains supplementary material available at 10.1038/s41598-025-08530-0.

## Introduction

Intrauterine adhesions occur at a high incidence after uterine trauma, seriously affecting female reproductive health. Currently, it is difficult to determine effective prevention methods for intrauterine adhesions, and the relevant mechanisms are still being investigated^1,2^. Estrogen is used to treat uterine trauma and may be beneficial to patients with intrauterine adhesion, regardless of the stage of the disease, but its role may be limited and require support from other adjuvant therapies^2,3^. Therefore, exploring the mechanism of endometrial restoration and identifying the key factors in estrogen-mediated damage repair may provide a reference for improving treatment options.

Tissue exposure following epithelial barrier disruption after the mechanical injury of the endometrium induces fibrin and fibroblast deposition, causing confluent growth and adhesions to the contralateral adjacent uterine wall^4,5^. Therefore, epithelial physical barrier reconstruction is important to avoid endometrial fibrosis and adhesion. Although in vitro transplanted stem cells may affect endometrial epithelial repair, they may have insufficient cell expansion or low survival rates^6,7^. Normally, the epithelium in endometrial reconstruction can originate from the proliferation of stem cells in the intact epithelial tissue adjacent to the injury; the stem cells in the endometrial stroma are also the source of unscarred epithelial remodeling^5,8,9^. Therefore, promoting the self-regeneration of epithelial stem cells, as well as differentiation or transformation of stromal cells in repair disorders may be a more effective way to reconstruct the epithelial barrier than using in vitro-derived cells.

S100A8 is an S100 member of the calcium-binding protein family, which is expressed in neutrophils, monocytes, endothelial cells, and epithelial cells; S100A8 expression is locally upregulated during inflammation^10,11^. S100A8/A9 heterodimer or its monomers can activate the repair function of stem cells to heal skin wounds, myocardial injury, and dental pulp injury by inducing angiogenesis, anti-inflammation, anti-fibrosis, and epithelial reconstruction^12–16^. S100A8/A9 alters the expressional fingerprint profile of mesenchymal stem cells (MSCs), such as differential genes *CCR7* and *IL-32*, which participate in promoting the migration and re-epithelialization of injured skin cells^12^. Overexpression of S100A8 in adipose-derived stem cells accelerates epithelial reconstitution of injured skin by promoting cell proliferation and differentiation^13^. In a previous study, we reported that S100A8 expression in fallopian epithelial cells was upregulated by estrogen^17^. It is related to the estrogen regulation of mucosal immune homeostasis and may participate in epithelial cell proliferation and differentiation^17^. Therefore, in this study, we aimed to confirm the effects and explore the mechanism of estrogen-regulated S100A8 during the reconstruction of the injured endometrial epithelium.

## Results

### Estrogen affected S100A8-positive immune cells and epithelial morphology of endometrium via S100A8 regulation

**Fig. 1:**
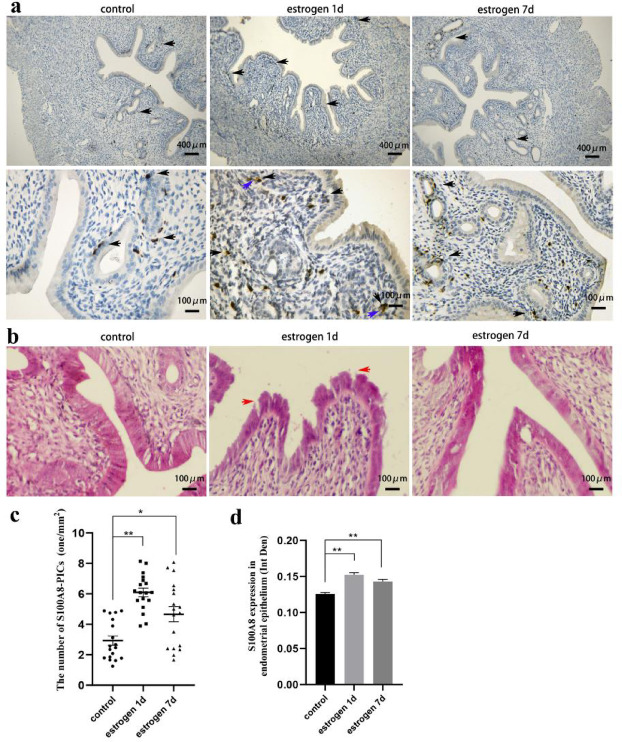
Effects of estrogen treatment on S100A8 expression in endometrium and epithelium morphology. (**a**) S100A8 immunohistochemical staining before and after estrogen treatment (1d/7d); black arrows indicate S100A8-positive immune cells (S100A8-PICs).The blue arrow indicates the release of S100A8 from S100A8-PICs. Scale bar is 100 or 400 μm. (**b**) Rat endometrial hematoxylin–eosin staining before and after estrogen treatment (1d/7d); red arrows show disrupted cell junctions. Scale bar is 100 μm. (**c**) S100A8-PICs number in the endometrium (1/mm^2^) before and 1 d/7d after estrogen treatment (n = 18, from six rats, the number was counted from three immunohistochemical images of each sample , mean ± SEM). (**d**) S100A8 expression in the epithelium before and 1 d/7d after estrogen treatment (n = 18, from six rats, the expression level was measured from three immunohistochemical images of each sample, mean ± SEM). * Results with *P* values < 0.05 were considered significant. ** Results with *P* values < 0.01 were considered very significant.

Immunohistochemical staining detected S100A8 expression and distribution of the S100A8-positive immune cells (S100A8-PICs) in the endometrium before and after estrogen treatment. One day after estrogen injection, S100A8-PICs were recruited from the gland to the stroma and epithelium, S100A8 was released (Fig. 1a). The number of S100A8-PICs in the endometrium significantly increased compared with that before estrogen administration (Fig. 1c). S100A8-PICs returned from endometrial stroma to the blood vessels or glands 7 days after estrogen injection (Fig. 1a), but the number of S100A8-PICs in endometrium did not change significantly compared with the estrogen 1d group (Fig. 1c). S100A8 was also expressed in the endometrial epithelium. S100A8 expression was significantly upregulated in epithelial tissues 1 day after estrogen injection (Fig. 1d), but the intensity of S100A8 expression still showed a considerable difference between epithelial cells and S100A8-PICs (Fig. 1a). The upregulated S100A8 in the epithelium appeared to decrease but was not significant 7 days after estrogen injection. Hematoxylin–eosin (HE) staining revealed that 1 day after estrogen injection, the epithelial interspaces were enlarged in all six samples, and the epithelial barrier was disrupted. However, the disruption of the six rats normalized 6 days after endometrial injury (Fig. 1a, b). The above results showed enhanced S100A8 expression in the epithelial tissue along with the recruitment of S100A8-PICs to the endometrial stroma and epithelium. The recruitment of S100A8-PICs disrupted the endometrial epithelial barrier, but the disruption was not continuously amplified by the release of S100A8.

### S100A8 influences S100A8-PICs reverse migration and accelerates structural reconstruction of injured epithelium

**Fig. 2:**
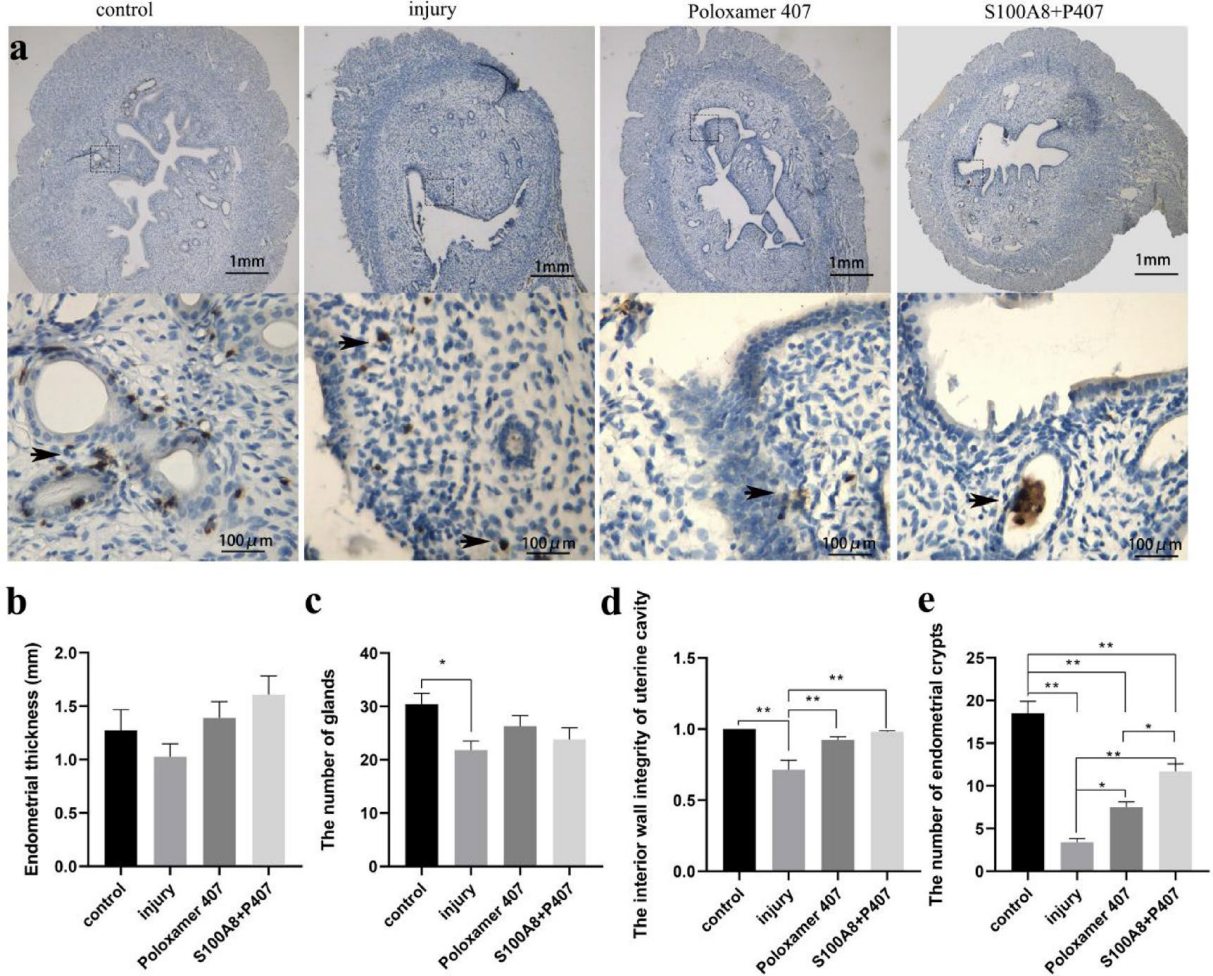
Effects of uterine cavity administration of S100A8 on the distribution of S100A8-PICs and endometrial morphology. (**a**) Distribution of S100A8-positive cells in the endometrium of four different groups with estrogen treatment (control, injury, P407, and S100A8 + P407 groups). Black arrows show S100A8-PICs. S100A8-PICs were distributed around the glands and blood vessels in the control group. S100A8-PICs in the site of injury or adhesion in the injury group and in the P407 group. S100A8-PICs reverse migrated to the glands or blood vessels in the S100A8 + P407 group. Scale bar is 100 μm or 1 mm. (**b**) Endometrial thickness in the four groups (n = 15, from 5 rats; 3 position was measured randomly in an image of each rat, mean ± SEM). (**c**) The number of endometrial glands in each group (n = 10, from 10 rats, glands were counted from a uterine horn cross-section image at 40 × of each rat, mean ± SEM). (**d**) The interior wall integrity of uterine cavity in each group (n = 10, from 10 rats, data was measured from a uterine horn cross-section image at 40 × of each rat, mean ± SEM). (**e**) The number of endometrial crypts in each group (n = 10, from 10 rats, crypts were counted from a uterine horn cross-section image at 40 × of each rat, mean ± SEM). * Results with *P* values < 0.05 were considered significant. ** Results with *P* values < 0.01 were considered very significant.

Immunohistochemical staining detected the distribution of endometrial S100A8-PICs in each group 6 days after endometrial injury and treatment while showing endometrial morphology. The rats in each group were injected with estrogen the day before injury to synchronize estrus and as a basic treatment for injury. The results showed that S100A8-PICs in the injury and P407 groups remained in the injured (injury group) or adhesion area (P407 group), whereas S100A8-PICs in the P407 + S100A8 group were mainly distributed in the glands or blood vessels and were rarely seen in the stroma (Fig. 2a). S100A8 appeared to influence the reverse migration of S100A8-PICs to the blood vessels or glands.

Uterine cavity morphology showed that the injury group still had a large area of unrepaired endometrium damage, incomplete epithelial tissue, and in-palace effusion; endometrium with columnar adhesion structures was found in the P407 group. However, the uterine cavity was in good shape, and the repair of endometrial epithelial tissue was observed in the P407 + S100A8 group (Fig. 2a). These results suggest that the effect of estrogen alone in promoting the repair of severely damaged endometrium is limited (at least within a week); however, S100A8 administration based on estrogen treatment is beneficial for endometrial repair. The measurements of different morphological indicators showed no significant differences in endometrial thickness between the groups (Fig. 2b), and the number of endometrial glands in the P407 and P407 + S100A8 groups was not significantly different from that of the injury group (Fig. 2c). The integrity of the interior wall of the uterine cavity in the P407 and S100A8 groups was significantly higher than that in the injury group. Although the difference between the P407 and S100A8 groups was not significant (Fig. 2d), a part of the damaged uterine interior wall in some rats was sealed by adhesion rather than by epithelial regeneration in the P407 group (Fig. 2a). Lumen epithelial evaginations form crypts, it forms synchronously with the glands^18^. Embryos implants in some of the crypts, ductal regions of glands continue to develop following embryos implantation^18^. The number of endometrial crypts in the P407 and P407 + S100A8 groups was significantly lower than that in the non-injured control group but significantly higher than that in the injury group, and the number of endometrial crypts in the P407 + S100A8 group was significantly higher than that in the P407 group (Fig. 2e). This finding indicates that S100A8 in the uterine cavity significantly promotes endometrial crypt formation in the injured endometrium.

### S100A8 induces the localization of proliferating cells from the stroma to the injured epithelium

**Fig. 3:**
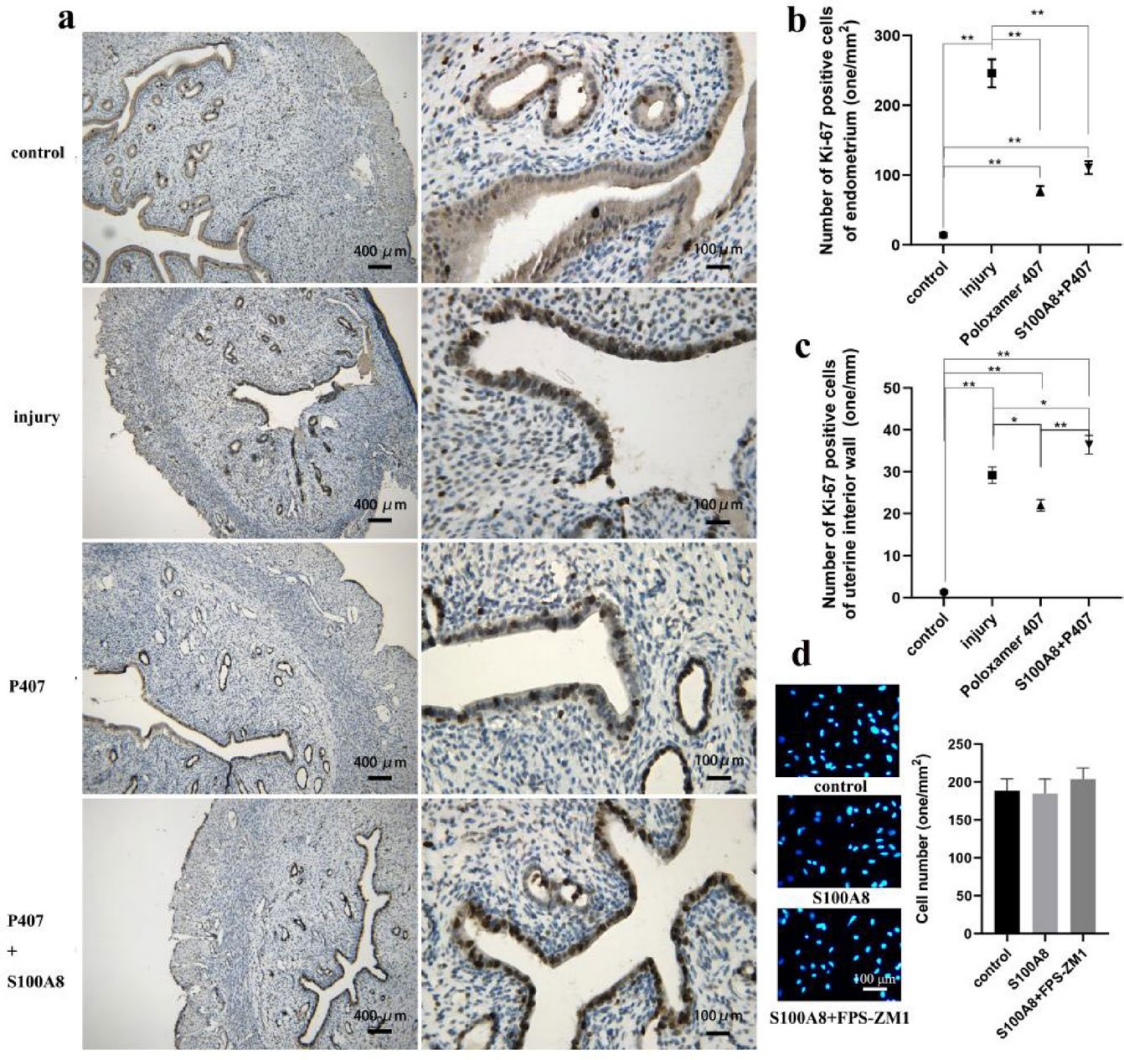
Effects of S100A8 on the distribution of Ki-67-positive proliferating cells in the uteri and the proliferation of endometrial cells. (**a**) Ki-67-positive proliferating cells were distributed in the endometrium or uterine interior wall in all groups (control, injury, P407, and S100A8 + P407 groups). Scale bar is 100 or 400 μm. (**b**) Number of Ki-67-positive cells in the endometrium of each group (n = 20, from ten rats, the number was counted from two immunohistochemical images of each sample, mean ± SEM). (**c**) Number of Ki-67-positive cells in the uterine interior wall of each group (n = 20, from ten rats, the number was counted from two immunohistochemical images of each sample, mean ± SEM). (**d**) Effects of S100A8 and its receptor inhibitor (FPS-ZM1) on the proliferation of cultured endometrial cells in vitro (n = 5, mean ± SEM). Scale bar is 100 μm. * Results with P values < 0.05 were considered significant. ** Results with P values < 0.01 were considered very significant.

Immunohistochemical staining detected the proliferation marker Ki-67 in the endometrium following injury or drug treatment. A comparison of the number of Ki-67-positive cells (one/mm^2^) in each group 6 days after injury revealed that the number of Ki-67-positive cells in the endometrium of each injury group (injury, P407, and P407 + S100A8 groups) was significantly higher than that in the control group, and the number of Ki-67-positive cells in the injury group was significantly higher than that in the other injured groups (Fig. 3a, b). This observation indicated that mechanical injury activated endometrial cell proliferation, the more damage exposure, the more cell proliferation in endometrium. The number of Ki-67-positive cells (one/mm) in the interior wall of the uterine cavity (including intact epithelium and deficient epithelium) in the three injured groups was still significantly higher than that in the control group. However, the number of Ki-67-positive cells in the interior wall of the P407 + S100A8 group was higher than that of the P407 and injury groups (Fig. 3c, very-significant or significant). Meanwhile, the Ki-67-positive proliferating cells in the P407 and P407 + S100A8 groups had a comparatively even distribution throughout the uterine wall, whereas those in the injury group at the site of severe epithelial injury could not cover the injury exposure (Fig. 3a). The number of Ki-67-positive cells in the interior wall of the P407 group was the lowest among the three injured groups, rather than injury group (Fig. 3c). That may be due to combined effects of less injury exposure and no S100A8 treatment in the P407 group. Primary endometrial cells were treated with S100A8 protein and its receptor inhibitor (FPS-ZM1), and cell density was visualized with 4′,6–2′-phenylindole dihydrochloride (DAPI) fluorescence staining. There were no significant differences in cell density among the control, S100A8, and S100A8 + FPS-ZM 1 groups, and S100A8 did not significantly promote endometrial cell proliferation (Fig. 3d).

### S100A8 promotes the formation of tight junctions in epithelial new migrants

**Fig. 4:**
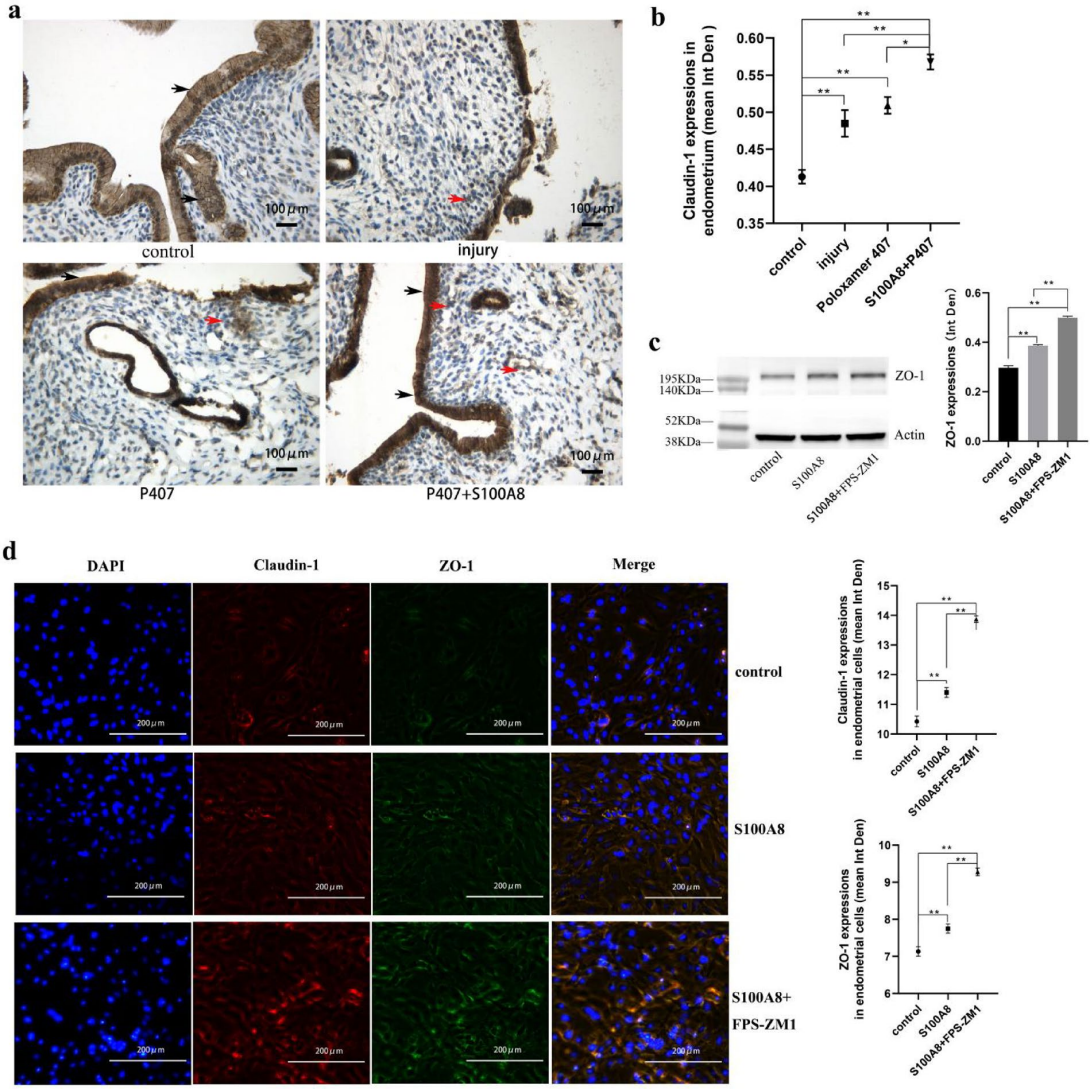
Effects of S100A8 on tight junctions of endometrial epithelium or endometrial cells. (**a**) Immunohistochemical staining showing Claudin-1 distribution in the endometrium of different groups (control, injury, P407, and P407 + S100A8 groups); black arrows show Claudin-1 accumulated below the cell membrane of the lateral contact zone between adjacent cells and red arrows show cells expressing Claudin-1 in the stroma. Scale bar is 100 μm. (**b**) Claudin-1 expression in the endometrium of different groups (n = 10, mean ± SEM). (**c**) Western blot showing ZO-1 expression in endometrial cells of three different groups (control, S100A8, and S100A8 + FPS-ZM1 groups, n = 3, mean ± SEM). Actin was used as the loading control. The full-length bands can be found in supplementary Fig. 1. The bands of ZO-1 and actin cropped from different parts of the same gel. (**d**) Immunofluorescence showing the co-expression of Claudin-1 (red) and ZO-1 (green) in the endometrial cells of the three groups (n = 5, mean ± SEM). The nucleus is stained blue. The scale bar is 200 μm. * Results with *P* values < 0.05 were considered significant. ** Results with *P* values < 0.01 were considered very significant.

The tight junction protein Claudin-1 is normally accumulated below the cell membrane of the lateral contact zone between epithelial cells. However, in the injury group, the integrity of epithelial tissue is difficult to maintain, and the tight junction protein Claudin-1 is scattered in the epithelial cells. Claudin-1 developed to varying degrees of reassembly at the lateral contact zone between the epithelial cells in all ten samples of the P407 and P407 + S100A8 groups, especially in the P407 + S100A8 group where it was orderly distributed (Fig. 4a).

Claudin-1 was expressed in the epithelial tissues; however, cells expressing low levels of Claudin-1 also appeared in stroma near the injured epithelium (Fig. 4a). Claudin-1 expression in the endometrium (including epithelium and stroma) of each injured group was significantly higher than that in the control group, indicating that injury promoted the expression of Claudin-1(Fig. 4a, b). Claudin-1 expression was significantly higher in the P407 + S100A8 group than that in the other injury groups (Fig. 4a, b). Primary endometrial cells were cultured in vitro and treated with S100A8 and its receptor inhibitor (FPS-ZM 1). Immunofluorescence and western blot (WB) analyses were performed to study the effect of S1000A8 on tight junction proteins in endometrial cells. The results showed that the expression and distribution of Claudin-1 and ZO-1 in the cells were synchronized (Fig. 4d). The expression of Claudin-1 and ZO-1 was significantly higher in the S100A8 group than in the control group; the expression of Claudin-1 and ZO-1 in the S100A8 + FPS-ZM 1 group continued to be higher than that in the S100A8 group (Fig. 4c, d). These results indicate that S100A8 promotes tight junction protein expression and orderly rearranges and accelerates the formation of tight junctions between the epithelial new migrants.

### S100A8 facilitates the transformation of stromal cells to supplement injured epithelial cells

**Fig. 5:**
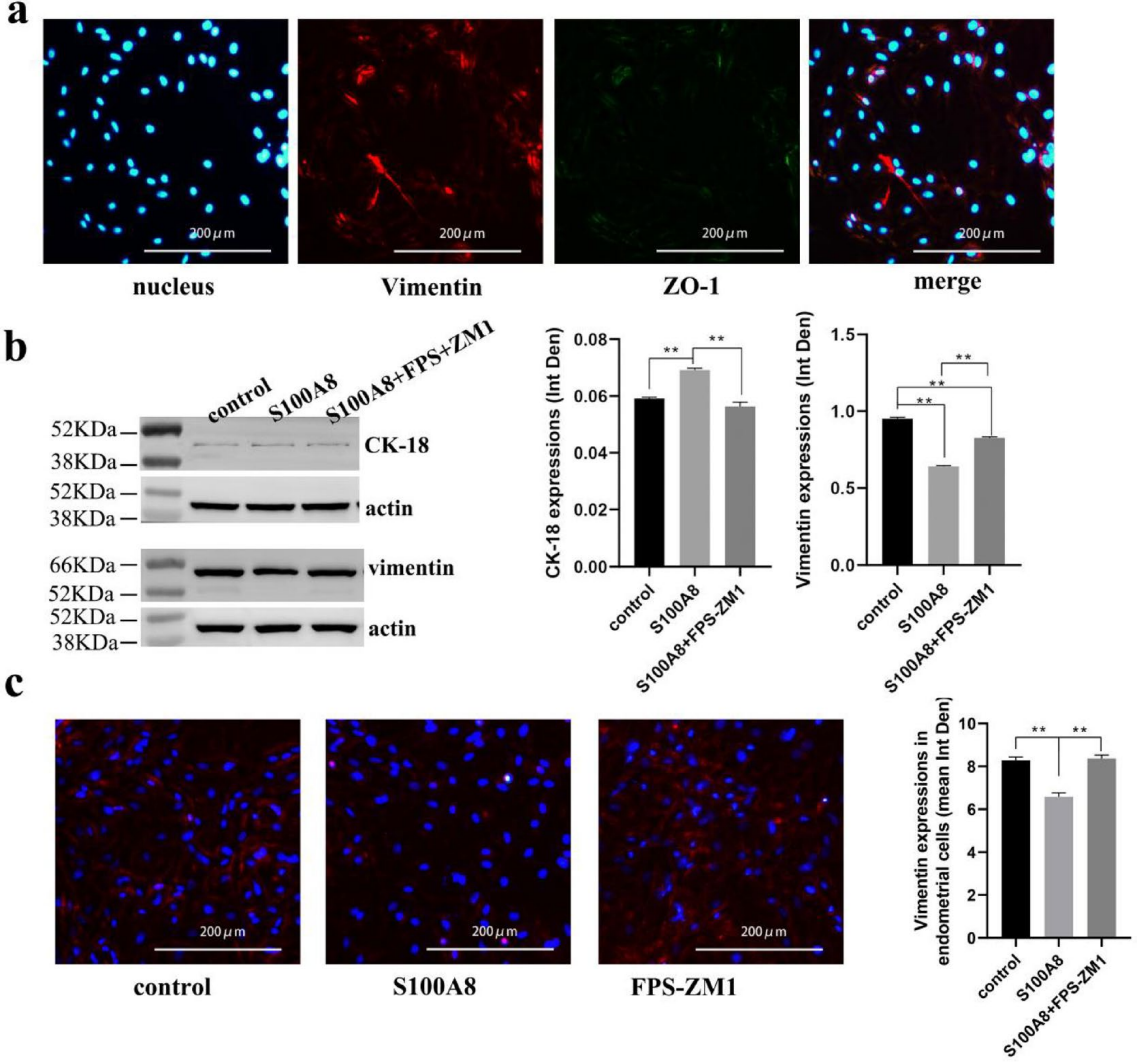
Effects of S100A8 on endometrial “double-featured” cells. (**a**) Vimentin (red) and ZO-1 (green) immunofluorescent double staining of cultured endometrial cells. Scale bar is 200 μm. (**b**) Western blot showing CK-18 and vimentin expression in endometrial cells from each group (control, S100A8, and FPS-ZM1 groups; n = 3, mean ± SEM). The full-length bands can be found in supplementary Fig. 1. Actin was used as the loading control. As the target proteins have a similar molecular weight to that of the loading control, the bands of CK18/vimentin and actin were cropped from different gels. (**c**) Immunofluorescence showing vimentin expression in endometrial cells of each group (n = 5, mean ± SEM). Scale bar is 200 μm. * Results with *P* values < 0.05 were considered significant. ** Results with *P* values < 0.01 were considered very significant.

The in vitro-cultured endometrial cells expressing Claudin-1 and ZO-1, as described in “S100A8 promotes the formation of tight junctions in epithelial new migrants” section, were not only epithelial cells. Immunofluorescence labeling of vimentin and ZO-1 in cultured endometrial cells showed that most cells expressed the stromal marker vimentin and epithelial marker ZO-1 synchronously, demonstrating “double features” (Fig. 5a). Endometrium injured in vivo also has such “double-featured” cells. Except the epithelial tissue, which highly expressed the epithelial marker Claudin-1, a small number of stromal cells near the injury site expressed low levels of Claudin-1 (Fig. 4a). Although its expression was very low, the WB analysis results showed that the expression of CK-18 in endometrial cells treated with S100A8 was significantly higher than that in the control and S100A8 + FPS-ZM1 groups (Fig. 5B). The expression of vimentin in the S100A8 group was the lowest and was significantly lower than that in the control and S100A8 + FPS-ZM 1 groups (Fig. 5b, c).

## Discussion and conclusion

Simulating estrous hormonal changes, estrogen has long been shown to indirectly recruit neutrophils to the reproductive tract by regulating the secretion of cytokines^19^. However, the cytokines that recruit neutrophils regulated by estrogen are still undefined. Our previous study showed that the expression of the cytokine S100A8 in epithelial cells was upregulated by estrogen after 7 h of treatment^17^. Cells release S100A8/A9, further recruiting the S100A8- and S100A9-rich neutrophils via Toll-like receptor 4 (TLR 4)^20^. The present study showed enhanced S100A8 expression in the epithelial tissue along with the recruitment of S100A8-PICs to the endometrial stroma and epithelium. This finding suggests that estrogen may indirectly recruit neutrophils by increasing S100A8 expression in the endometrial epithelium. However, immunocyte recruitment is associated with epithelial damage. After performing the defensive functions, chemotactic neutrophils can cause chronic damage if they remain in the tissue, but could regulate tissue damage by reverse migration, cell apoptosis, secretory vesicle release, neutrophil extracellular trap (NET) release, or other ways^21–23^. S100A8 and S100A9 is released by NET formation^24^. S100A8 and S100A9 may continue to amplify neutrophil recruitment but may also be related to the damage-regulating effects of neutrophils^21–25^. Our study showed that transient damage to the normal endometrial epithelium under estrogen treatment was repaired after 6 days, S100A8 released by recruited S100A8-PICs did not amplify damage step-by-step, and S100A8-PICs returned to the glands or blood vessels. S100A8 released by neutrophils may have damage-regulating effects. Therefore, an endometrial injury model may be used to analyze the associations.

Intrarectal calprotectin (S100A8/A9) protects against colitis, especially in the mucosal epithelium, and calprotectin suppresses inflammatory factor expression and reduces the neutrophil number in the blood^25^. Research on infected chronic wound healing indicated reduced proinflammatory responses after S100A8/A9 injection favored wound healing when blood neutrophil numbers decreased and local expression of interferon-γ was reduced^26,27^. Our results also showed that S100A8 in the uterine cavity significantly accelerated endometrial repair, influenced the reverse migration of S100A8-positive cells into the glands or blood vessels. These results do not necessarily contradict the chemotactic effect of S100A8. The chemotactic and anti-inflammatory effects may be exerted in different stages of the immunomodulation of S100A8. When S100A8 is secreted by epithelial cells regulated by estrogen or released from injured epithelial tissues, S100A8 recruits neutrophils to perform the defense functions^19,20^. Subsequently, the recruited neutrophils are further induced by estrogen and release high concentrations of S100A8 and S100A9, which feedback to regulate inflammation and neutrophil recruitment^23,25,28^. Auto-inhibition of S100A8/A9 aggregation prevents undesirable systemic effects. Calcium-induced (S100A8/A9)_2_ tetramer formation restricts neutrophil activity by hiding the TLR4/MD2- binding site^29,30^. In addition to auto-inhibition, the S100A8/A9 tetramer can also limit monocyte adhesion and migration through the CD69 receptor^31^. However, if the release of S100A8 and S100A9 is insufficient for feedback regulation, immune cells may remain, or progressive compensatory chemotaxis leads to poor chronic wound healing or acute injury^26,27,32^. Therefore, when the endometrium is seriously damaged, the S100A8 regulatory function affected by estrogen is limited, the damage heals slowly, and supplementation of S100A8 in the uterine cavity is required. S100A8 not only regulates neutrophil reverse migration to reduce epithelial damage but also promotes crypt formation in the injured endometrial epithelium. Locally enhanced proliferation in the lumen epithelium, which induces tissue buckling, apical evagination, crypt outgrowth, and embryo implants after crypt formation^18,33^. Therefore, S100A8 may promote epithelial cell proliferation or proliferating cell localization to the epithelium to form endometrial crypts.

Injury could activate cell proliferation through blood exposure, inflammatory factors, extracellular matrix, and so forth. Failure to heal led to notable endometrial cell proliferation in the injury group; the more damage exposure, the more cell proliferation in endometrium. Although the number of Ki-67-positive cells in endometrium was the highest in the injury group, their number in uterine interior wall was the highest in the P407 + S100A8 group, indicating that proliferating cells tended to be localized to the epithelium rather than to the stroma in the P407 + S100A8 group. Although epithelial stem cell proliferation can repair the damaged epithelium, the migration and transformation of specific subsets of stromal cells into the /epithelium could cover the exposure and serve as a source for epithelial reconstruction^8,9^. Our results showed that S100A8 did not significantly promote endometrial cell proliferation. Basu et al.^12^ found that S100A8/A9 upregulates the expression of genes involved in the recruitment of endogenous mesenchymal stem cells (MSCs) and re-epithelialization and significantly promotes the repair ability of MSCs on the epithelial tissues of skin wounds. Therefore, S100A8 in the uterine cavity may not directly induce epithelial cell proliferation but more likely promotes the accumulation of mesenchymal cells activated by mechanical injury to the injured epithelium.

Studies on chronic rhinosinusitis have detected low expression of S100A8 and S100A9 in the epithelial cells of patients with chronic rhinosinusitis, which may lead to the destruction of the epithelial physical barrier and poor repair ability^34–36^. However, the studies on the benefits of S100A8 and S100A9 for the formation of epithelial physical barriers are rarely reported. Our research showed that S100A8 in the uterine cavity promotes the aggregation of Claudin-1 in the lateral contact zone between adjacent epithelial cells and increases the expression of the tight junction proteins Claudin-1 and ZO-1 in endometrial cells. These results provide evidence that S100A8 aids physical barrier reconstruction of the endometrial epithelium. RAGE is a classical receptor for S100A8, but the two do not correspond one-to-one. RAGE may have physiological roles in promoting the proliferation or differentiation of normal alveolar epithelial cells, but it is also believed to be involved in the pathological process of fibrosis, with its differing functions potentially being ligand-related^37^. FPS-ZM1 is an inhibitor of RAGE receptor. Unexpectedly, FPS-ZM 1 did not inhibit the promoting effect of S100A8 on tight junction proteins but elevated the tight junction protein expression in endometrial cells, suggesting that S100A8 regulation of tight junctions may be independent of RAGE receptors . Furthermore, FPS-ZM1 may have inhibited the RAGE-mediated fibrotic process, thereby acting synergistically with S100A8 to promote epithelial reconstruction.

Mesenchymal cells in the endometrial stroma have a high regenerative potential, and endometrial epithelial regeneration can be derived from stromal cells^9,38^. Single-cell sequencing analysis of postmenstrual endometrial repair revealed that stromal cells expressing epithelial cell markers reflect the transformation and transition of stromal cells to epithelial cells^9^. In the present study, endometrial stromal cells simultaneously expressed epithelial markers when injured in vivo or cultured in vitro. Such “double-featured” cells may be the transition cells, formed when endometrial stromal cells are transformed to epithelial cells. S100A8 and S100A9 are located in the epidermal differentiation complex of human chromosome 11 and are important genes involved in epithelial differentiation and regeneration^34^. In colon cancer, the infiltration of S100A8 + cells into the stroma is an innate immune response that restrains epithelial-mesenchymal transition (EMT), suggesting a role for S100A8 in the maintenance of epithelial differentiation^39^. In the present study, S100A8 increased the expressions of ZO-1 and CK-18 and simultaneously decreased the expression of vimentin in "double-featured" cells. As cytoskeleton and tight junction proteins favor the formation of epithelial cell morphology and polarity, S100A8 may facilitate the transformation of cells with “double features” into epithelial cells. However, our study results cannot exclude that these transition cells may be derived from the epithelium. Further evidence is required to determine whether S100A8 restricts EMT or promotes mesenchymal-epithelial transition (MET) and verify downstream S100A8-activated signaling pathways.

In conclusion, S100A8 expression, regulated by estrogen, is a key factor affecting the efficiency of estrogen-mediated endometrial damage repair. After significant endometrial damage, the ability of estrogen to regulate inflammation and damage via self-S100A8 is limited. However, S100A8 administration after estrogen treatment significantly promoted endometrial epithelial integrity and crypt regeneration. S100A8 regulation in the reverse migration of S100A8-PICs could prevent the sustained damage. Meanwhile, S100A8 promotes the formation of tight junctions between the epithelial new migrants and may facilitate the transformation of cells derived from endometrial stroma into epithelial cells.

## Materials and methods

### Estrogen treatment

Six 8–9-week-old female Wistar rats were intramuscularly injected with estradiol benzoate (0.5 mg/kg). The rats were euthanized by injecting an overdose of pentobarbital sodium before uterine collection. At 1 and 7 days after injection, the uterus was fixed, sectioned, and stained. Estrogen injections were used to synchronize estrus and as estrogen treatment affecting a healthy uterus.

The animal study protocols were implemented in accordance with the “Guidelines for Euthanasia of Experimental Animals T/CALAS 31–2017” (issued by the Chinese Association for Laboratory Animal Science, Beijing, China) and the “Guide for the Care and Use of Laboratory Animals” (published by the National Academy of Sciences, Washington, D.C., USA). All methods are reported in accordance with the ARRIVE guidelines for animal research (https://arriveguidelines.org). The rats were purchased from Beijing Vital River Laboratory Animal Technology Company (SPF, Beijing, China).

### Rat model of endometrial injury and intrauterine drug administration

Thirty-six female Wistar rats aged 8–9 weeks were intramuscularly injected with estradiol benzoate (0.5 mg/kg) 1 day before surgery to synchronize estrus and estrogen treatment affecting injury repair. The rats were randomly divided into three groups using a computer-based random order generator: injury, P407, and P407 + S100A8 groups. Poloxamer 407 (P407) is a non-ionic surfactant that maintains its solution form at room temperature and transforms into a gel at body temperature. It is commonly used as a drug carrier to prolong the residence time of drugs at the site of administration, which helps improve bioavailability and therapeutic effects. The left uterine horns of each group were used for the drug treatment and as an injury model, whereas the right uterine horns were used as a control. Before surgery, rats were intraperitoneally injected with 15–40 mg/kg pentobarbital sodium for inducing anesthesia. Rats of the injury group were subjected to a 1-cm incision in the left back, an incision was made in the ovarian proximal end of the uterus, the inner wall of the uterus was scratched with a pair of ophthalmic tweezers, the incision was stitched, and 200 μL phosphate buffered solution (PBS) was injected into the uterus. Based on previous injury, rats of the P407 group were injected with 200 μL Poloxamer 407 (HY-D1005, MEC, USA), prepared at a concentration of 0.2 g/mL using PBS, into the uterine cavity, and the incisions were closed after 1–2 min of gel solidification. Based on previous injury, rats of the P407 + S100A8 group were injected with 200 μL Poloxamer 407 carrying S100A8 recombinant protein (HY-P71275, MCE, USA), prepared at a concentration of 5 µg/mL using Poloxamer 407 liquid, into the uterine cavity, and the incisions were closed 1–2 min after gel solidification. Postoperatively, the rats were administered antibiotics. The rats were euthanized by injecting an overdose of pentobarbital sodium before uterine collection. The uteri of rats from each group were collected 7 days after estrogen injection for sectioning and staining. We determined the number of rats per group by considering the test type, significance, the power of the experiment, and the operation time. Rats were excluded if they died prematurely. One rat of injury group and 2 rats of P407 group died caused by postoperative infection of self-biting the wound. Therefore, in order to unify the sample size, the data on uterine mechanical injury were actually based on 10 animals per group.

### Immunohistochemical staining

The uterus was fixed in 4% paraformaldehyde (P0099; Beyotime, Shanghai, China), embedded in paraffin, and sectioned (5–7 µm). The paraffin-embedded sections were deparaffinized and rehydrated. Antigen retrieval was performed using citric acid (pH 6.0) for 15 min, followed by cooling to room temperature. Endogenous peroxidase activity was blocked using 3% hydrogen peroxide and incubated at room temperature in the dark for 25 min. The tissues were sealed with 3% goat serum (C0265,; Beyotime, Shanghai, China) for 30 min at 25 ℃. The tissues were incubated with primary antibodies (1:1000, MRP8 rabbit polyclonal antibody, GB11421; 1:1000, Ki-67 rabbit polyclonal antibody, GB111499; 1:1000, Claudin1 mouse monoclonal antibody, GB12032; Servicebio, Wuhan, China) overnight at 4 °C, and then incubated with the secondary antibodies (1:2000, Horseradish peroxidase-conjugated goat anti-rabbit/rat IgG, GB23302/23,030; Servicebio, Wuhan, China) at room temperature (25℃) for 1 h. Diaminobenzidine (DAB) (P0202; Beyotime, Shanghai, China) was used for the chromogenic reaction. Nucleus staining, dehydration, and mounting were performed. Optical microscope (OLYMPUS, Japan) and processing software (MIE 3.1, China) were used to acquire the images.

### Culture of endometrial cells

The uteri of 3–5 rats were collected sterile, dissected longitudinally, and digested in trypsin at 37 °C for 20 min. The uterine wall was scraped using a cell scraper. After collection, the cells were washed several times and cultured in separate flasks at a concentration of 10^6^ in DMEM/F12 cell culture medium (C11330500BT; Gibco, Shanghai, China) with 20% fetal calf serum (80,230–6412; Every Green, Hangzhou, China), and cells from 2–4 passages were used for subsequent studies. The cultured cells were randomly divided into three groups according to the experimental design: the control, S100A8 (0.5 µg/mL, HY-P71275; MCE, Shanghai, China), and S100A8 + FPS-ZM 1 (5 µM, HY-19370; MCE, Shanghai, China) groups.

### Immunofluorescence

Primary endometrial cells grown on cover glasses were fixed in acetone, treated with 1% Triton-X 100 (P0096; Beyotime, Shanghai, China), and labeled with primary antibodies (1:1000, anti-ZO-1 rabbit polyclonal antibody, GB111402; 1:1000, anti-Claudin1 mouse monoclonal antibody, GB12032; 1:1000, anti-Vimentin mouse monoclonal antibody, GB12192; Servicebio, Wuhan, China) and secondary antibodies (1:100, goat anti-rabbit IgG/FITC antibody, GB22303; 1:100, goat anti-mouse IgG/Cy3 antibody, GB21301; Servicebio, Wuhan, China). Fluorescence microscopy (Nikon, Japan) and processing software (NIS-Elements, Japan) were used to observe the labeled cells and acquire the images.

### WB analysis

WB analysis was performed as described previously^17^, using primary antibodies (1:1000, anti-ZO-1 rabbit polyclonal antibody, GB111402; 1:1000, anti-Vimentin mouse monoclonal antibody, GB12192; 1:1000, anti-Cytokeratin 18 mouse monoclonal antibody, GB15232; Servicebio, Wuhan, China) and the secondary antibodies (ready for use, S-vision immunohistochemistry multimeric secondary antibody mouse/rabbit common, G1303; Servicebio, Wuhan, China). Actin was detected as a loading control. The images of WB were acquired using a chemiluminescence imaging system (Servicebio, China).

### Statistical analysis and data quantification

The GraphPad Prism software (version 8.0; GraphPad Software, San Diego, CA, USA) was used for generating figures and for performing statistical analysis of significant differences between the groups. Experimental data were tested for homogeneity of variance and normality. The one-way analysis of variance was used to test the significance of differences between groups (ordinary ANOVA test or Brown–Forsythe and Welch ANOVA test, alpha level = 0.05, *P < 0.05, **P < 0.01). The Image J software (version 1.34j; National Institutes of Health, Bethesda, MD, USA) was used to measure the integrated densities of the immunohistochemical images, immunofluorescence images, and WB images. The protein expression level of the target gene was normalized with that of the loading control for the WB analysis.

The total number of endometrial S100A8-PICs were manually counted using three immunohistochemical images of each sample in all groups. The endometrial area was measured for calculating the number of S100A8-PICs per square millimeter. Three images was randomly taken from the immunohistochemical staining uterine horn cross-section of each sample in all groups at 400 × magnification. The mean integrated densities of the epithelium in the image were measured to represent the S100A8 expression level. The endometrial thickness were measured from three uterine horn cross-section image (100 ×) of each sample in different groups, and one position was taken to measure randomly in each image. The glands were counted from a complete uterine horn cross-section image of each sample in different groups. Including both closed glands and those open to the uterine cavity (crypt) in the endometrium which marked by immunohistochemical staining of Claudin-1.

## Electronic supplementary material

Below is the link to the electronic supplementary material.

Supplementary Material 1

Supplementary Material 2

Supplementary Material 3

Supplementary Material 4.

## Data Availability

The original contributions presented in the study are included in the article/supplementary material, further inquiries can be directed to the corresponding author/s.
