# Supplementary figures and images for: S100A8 regulated by estrogen improves injured endometrial epithelium reconstruction by promoting tight junction formation and stromal cell transformation
Source: Sci Rep. 2025 Jul 1;15:21506. doi: 10.1038/s41598-025-08530-0 (PMC12219834; doi:10.1038/s41598-025-08530-0)

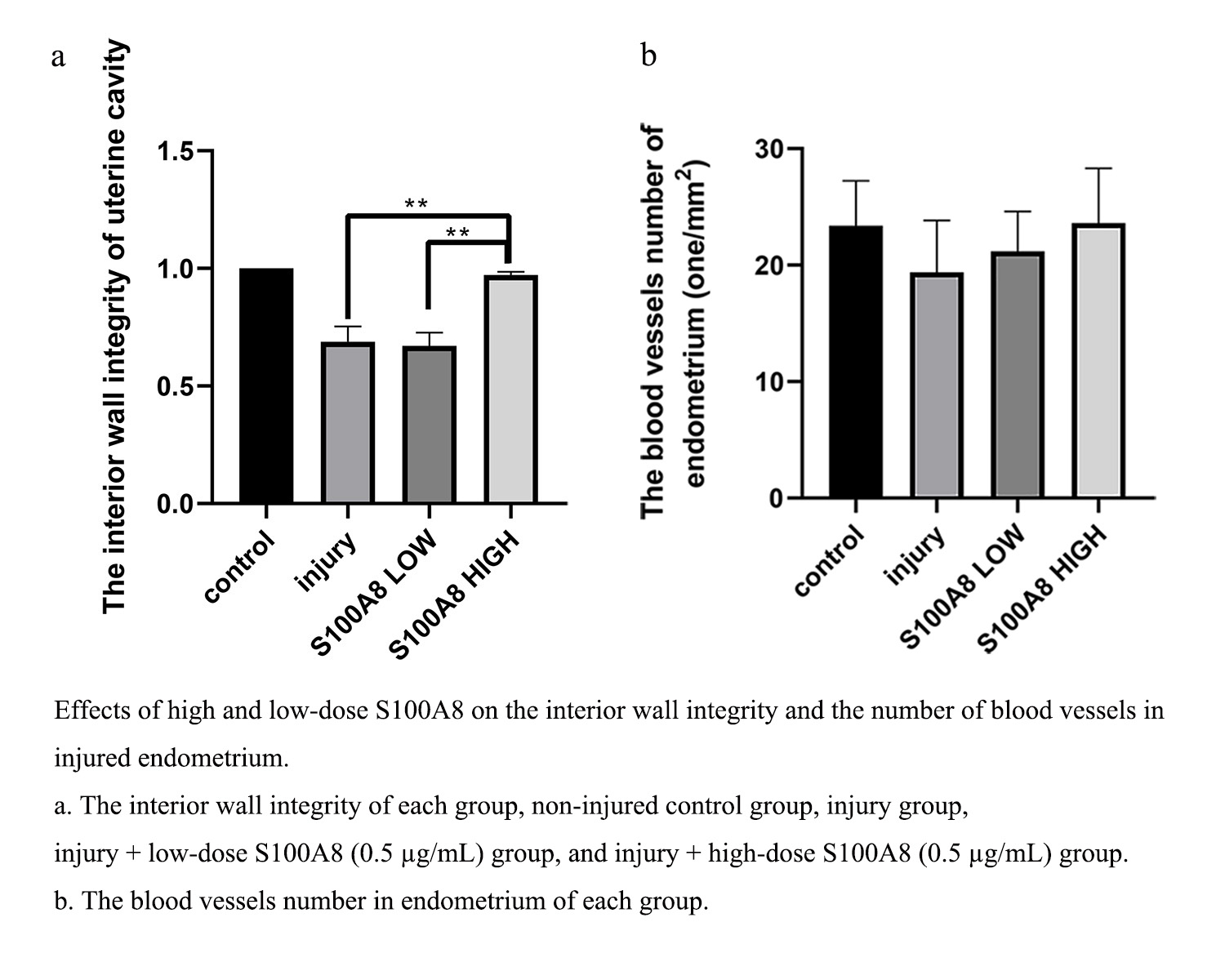

Supplement: Supplementary file 1 — Supplementary Material 1 [file 41598_2025_8530_MOESM1_ESM.jpg]

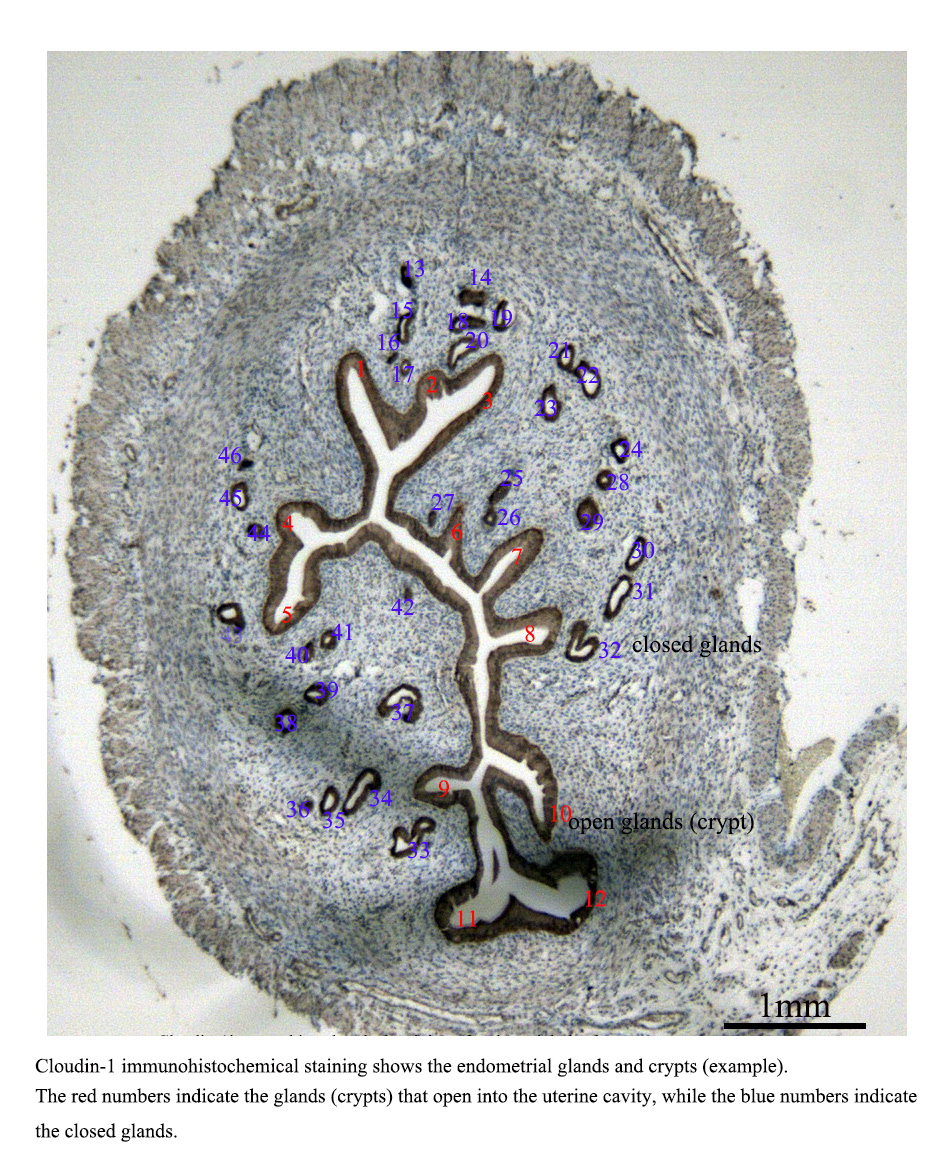

Supplement: Supplementary file 2 — Supplementary Material 2 [file 41598_2025_8530_MOESM2_ESM.jpg]

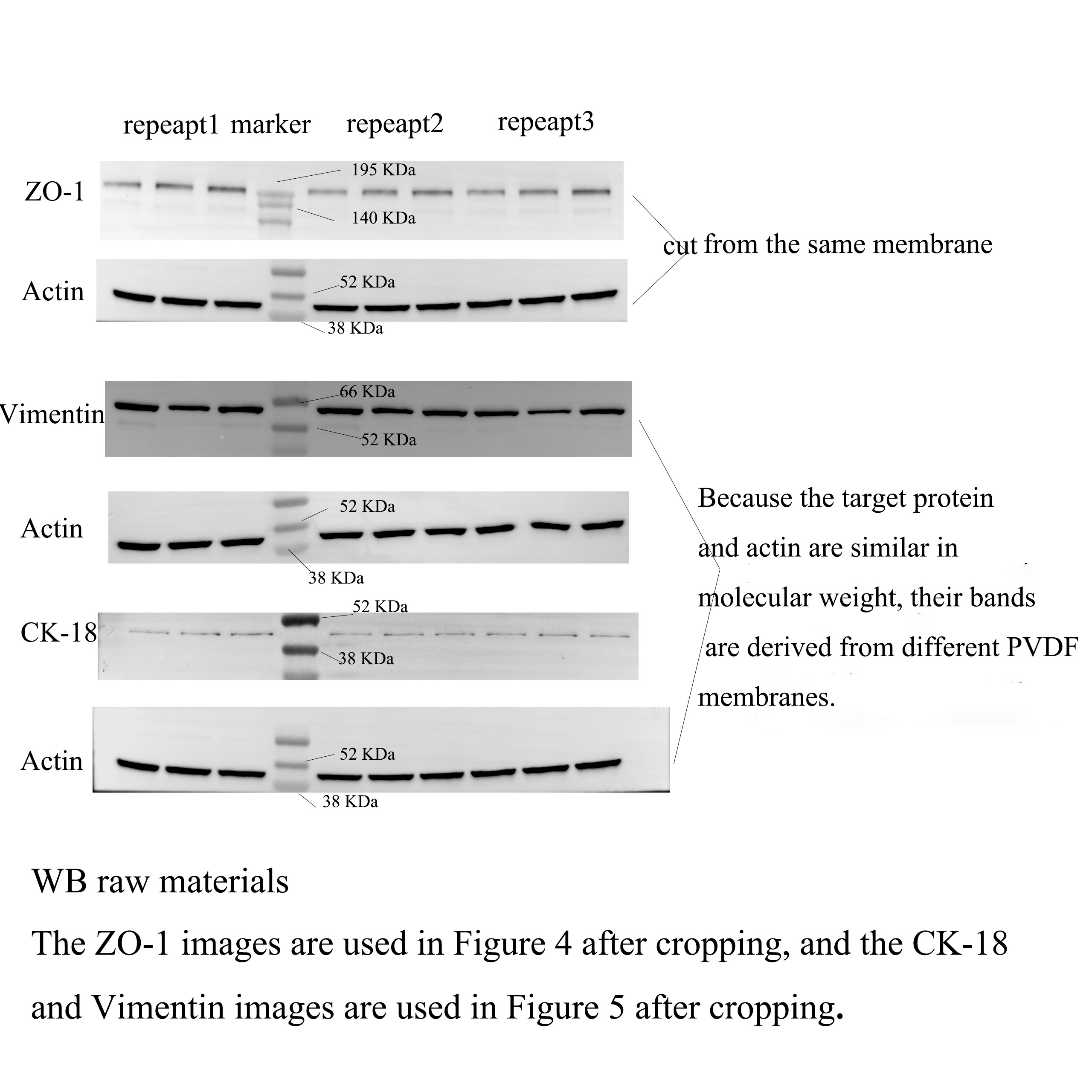

Supplement: Supplementary file 3 — Supplementary Material 3 [file 41598_2025_8530_MOESM3_ESM.jpg]
